# BZR1 and BES1: From Brassinosteroid Signaling Termini to Multifunctional Integrators of Plant Growth and Environmental Adaptation

**DOI:** 10.3390/ijms27219434

**Published:** 2026-10-22

**Authors:** Fengxiao Guo, Shenghong Ge, Mingguang Lei

**Affiliations:** College of Life and Environmental Sciences, Hangzhou Normal University, Hangzhou 311121, China; 2024112010021@stu.hznu.edu.cn

**Keywords:** BZR1/BES1, brassinosteroid signaling, post-translational modifications, transcriptional regulation, nutrient signal integration, abiotic stress responses, crop improvement

## Abstract

BZR1 (Brassinazole-resistant 1) and BES1 (bri1-EMS suppressor1) are the principal transcription factors that convert brassinosteroid (BR) perception into genome-wide transcriptional programs governing plant growth, development, and stress responses. Once regarded as end-effectors of a linear receptor-to-nucleus relay, they are now recognized as central hubs that integrate diverse endogenous and environmental cues into context-specific transcriptional outputs. We synthesize recent progress across four connected levels. Structurally, a modular architecture enables simultaneous target recognition, signal input, and output control. Mechanistically, the canonical BIN2-mediated phosphorylation switch is expanded by upstream regulators that couple membrane dynamics, carbon status, protein homeostasis, and nuclear import to BZR1/BES1, while ubiquitination, SUMOylation, redox modification, and acetylation form an interconnected post-translational network. During transcriptional execution, dephosphorylated BZR1/BES1 partition targets into activated and repressed sets through cis-element preference, corepressor recruitment, chromatin modifiers, and partnerships with factors such as PIF4 and ARF6. Physiologically, BZR1/BES1 coordinate anutrient-responsive metabolism, yield-related traits, and abiotic-stress tolerance, with marked functional divergence among crop orthologs. We highlight open questions in signal-integration specificity and discuss how BZR1/BES1 modules could be engineered for climate-resilient crops.

## 1. Introduction

Brassinosteroids (BRs) are steroidal phytohormones that regulate almost every phase of plant growth and development, from cell elongation and vascular differentiation to photomorphogenesis, reproduction, and stress adaptation [1,2]. Appropriate BR signaling output allows plants to realize their intrinsic growth potential and to adjust to fluctuating environments, whereas disruption of the pathway causes severe dwarfism, reduced fertility, and heightened stress sensitivity [3,4]. At the terminus of this pathway, the transcription factors BZR1 and BES1 act immediately downstream of the BR signal and directly activate or repress thousands of target genes [5,6]. Understanding how BZR1/BES1 are regulated, and how they in turn regulate the genome, is therefore central to explaining how BR signals drive growth and environmental adaptation.

Decades of work have produced a coherent core model of BR signal transduction. When BR is abundant, it binds the leucine-rich-repeat receptor kinase BRI1 (Brassinosteroid-Insensitive 1) and inactivates the GSK3-like kinase BIN2 (Brassinosteroid-Insensitive 2). Dephosphorylated BZR1/BES1 then accumulate in the nucleus and reprogram transcription [7,8]. When BR is limiting, active BIN2 phosphorylates BZR1/BES1, promoting their retention in the cytoplasm by 14-3-3 proteins and their turnover through the ubiquitin–proteasome system [9,10]. In this framework, BZR1/BES1 activity is set primarily by the intracellular BR concentration. This model has guided the field for two decades, but it does not fully explain the functional breadth of BZR1/BES1 in complex, fluctuating environments.

Recent studies have pushed well beyond this linear scheme in two directions. First, a series of upstream regulators that act directly on BZR1/BES1, rather than through BR concentration, have been identified. These regulators couple membrane–protein dynamics, carbon status, protein-folding homeostasis, and nucleocytoplasmic transport to BZR1/BES1 activity [11,12,13,14]. Second, the precision of BZR1/BES1 control has been reinterpreted through the lens of post-translational modification (PTM). Superimposed on the classical phosphorylation switch, ubiquitination, SUMOylation, and redox modification form interdependent regulatory layers that jointly tune protein stability, subcellular localization, and transcriptional activity [15]. The synergy and antagonism among these modifications convert BZR1/BES1 regulation from a binary on/off logic into a multidimensional, tunable integration system.

Functionally, BZR1/BES1 now extend far beyond the control of cell elongation. They participate in the sensing and metabolic integration of multiple nutrients, including carbon, nitrogen, phosphorus, sulfur, and boron [13,16,17,18,19]. They shape developmental and yield-related traits such as plant architecture, fruit ripening, pollen development, and flowering time [20,21,22,23]. They also confer tolerance to abiotic stresses including salinity, drought, and temperature extremes [14,24,25,26]. Critically, by combining growth-promoting and stress-responsive inputs, BZR1/BES1 act as molecular switches that arbitrate growth–defense trade-offs [21,27]. Together, these advances mark a conceptual shift. BZR1/BES1 have evolved, in our understanding, from terminal effectors of the BR pathway into multi-signal integration hubs.

**Figure 1:**
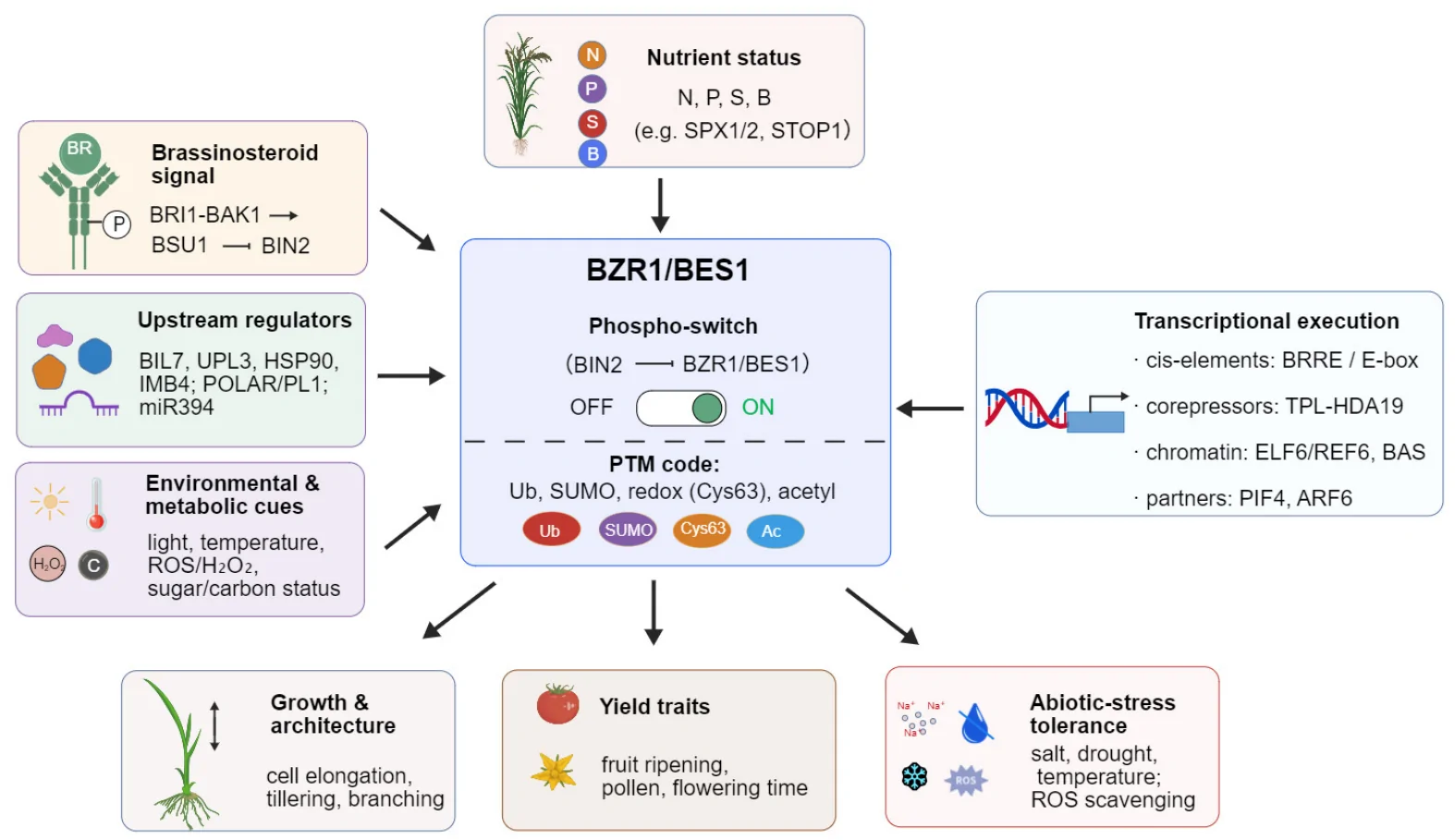
BZR1/BES1 function as a multi-signal integration hub. Diverse inputs converge on dephosphorylated BZR1/BES1. These inputs include the brassinosteroid (BR) signal relayed through BRI1–BAK1 and the BSU1-mediated inactivation of BIN2, direct regulators of the transcription factors (BIL7, UPL3, HSP90, IMB4), scaffold and microRNA modules (POLAR/PL1, miR394), environmental and metabolic cues (light, temperature, ROS/H_2_O_2_, sugar/carbon status), and nutrient status (N, P, S, B). Their activity is set by a phosphorylation switch overlaid with a post-translational code (ubiquitination, SUMOylation, redox modification of Cys63, and acetylation). Through cis-element choice (BRRE versus E-box), corepressors (TPL–HDA19), chromatin modifiers (ELF6/REF6, BAS), and combinatorial partners (PIF4, ARF6), the hub is translated into context-specific transcriptional programs that govern growth and plant architecture, yield-related traits, and abiotic-stress tolerance. Diagram templates were adapted from BioGDP (https://biogdp.com) [28].

This review synthesizes recent progress on the BZR1/BES1 family along the logical axis of the proteins themselves. We begin with their molecular architecture, and then cover the multilayered control of their activity by phosphorylation, upstream regulators, and PTM crosstalk then the mechanisms by which activated BZR1/BES1 execute differential transcription, and finally their physiological outputs in nutrition, development, and stress, together with their functional divergence across crops. Drawing these threads together, we frame BZR1/BES1 as a multi-signal integration hub and consider how this view can be exploited to improve crop productivity, nutrient-use efficiency, and stress resilience under climate change and resource constraints (Figure 1).

## 2. Molecular Architecture of the BZR1/BES1 Family

### 2.1. Discovery, Nomenclature, and Family Membership

BZR1 and BES1 were identified independently in two Arabidopsis genetic screens. BZR1 emerged from a screen for resistance to the BR-biosynthesis inhibitor brassinazole, whereas the gain-of-function bes1-D allele was recovered as a suppressor that partially restored the phenotype of the weak BR-receptor mutant *bri1* [29,30]. Both proteins act as master transcription factors that activate or repress thousands of BR-responsive genes and thereby mediate the bulk of BR signaling output [5,6].

BZR1 and BES1 belong to a small, highly conserved, plant-specific family. In Arabidopsis, this family comprises six members, namely BZR1, BES1, and the four BES1/BZR1 homologue proteins BEH1–BEH4. Despite strong sequence similarity, the members have diverged in expression pattern, post-translational regulation, and physiological function [31,32,33]. BZR1 and BES1 dominate the canonical BR response, whereas the BEH proteins tend to act in more restricted developmental contexts, sometimes with partially redundant or even antagonistic roles [32,33]. This combination of conservation and functional specialization provides the molecular substrate for the family’s capacity to serve as a multi-signal integration hub.

### 2.2. Modular Domain Organization

BZR1/BES1 proteins are strikingly modular. From N- to C-terminus, they comprise a DNA-binding domain, a central regulatory region rich in GSK3-like (BIN2) phosphorylation sites and containing a PEST motif, and a C-terminal regulatory/interaction domain [32]. This layout lets the proteins recognize specific cis-elements through the N-terminus while dynamically adjusting their nucleocytoplasmic distribution, stability, and transcriptional activity through modifications in the central and C-terminal regions [34,35], so that a single protein can act both as a terminal effector of BR signaling and as an integration point for diverse environmental inputs [1].

The N-terminal DNA-binding domain is an atypical, plant-specific basic helix–loop–helix (bHLH) module that recognizes the BR-response element BRRE (CGTGT/CG) and the E-box (CANNTG) in target-gene promoters [36]. BZR1 binds BRRE efficiently on its own and largely represses BR-response genes through this element [37], whereas BES1 typically heterodimerizes with the bHLH factor BIM1 to bind E-boxes and activate transcription [38]. Structural analysis showed that the BZR1 DNA-binding domain forms an atypical bHLH dimer that recognizes the shape of the DNA backbone as much as specific bases, achieving sequence-specific binding to the 5′-CGTG-3′ core [39]. This shape-readout mechanism helps explain how a compact domain can discriminate between closely related elements.

The central regulatory region carries numerous conserved Ser/Thr residues phosphorylated by BIN2 [1]. BIN2-dependent phosphorylation has a dual effect. In the cytoplasm, it promotes 14-3-3 binding and cytoplasmic retention [10], while in the nucleus, it directly suppresses DNA-binding activity [7,40]. The adjacent PEST motif (rich in proline, glutamate, serine, and threonine) is a key determinant of protein stability and is required for the interaction between the phosphatase PP2A and BZR1, and thus for BZR1 dephosphorylation [15,35]. This region is therefore the principal interface at which external signals enter the protein, and where phosphorylation, ubiquitination, and SUMOylation converge.

The C-terminal domain is the most divergent among family members and mediates homo- and heterodimerization as well as transcriptional-activity control. In maize, the C-terminus of ZmBES1/BZR1-5 contains a BAM domain, and homodimerization through this domain is required for transcriptional activity [36]. In Arabidopsis, the C-terminus of BZR1/BES1 mediates physical interaction with the corepressor TOPLESS (TPL) and the histone deacetylase HDA19, extending transcriptional control to the level of chromatin modification [41,42]. The C-terminus also contains a docking motif required for BIN2 to bind and phosphorylate its substrates [43]. The diversity of C-terminal sequences is thus a plausible structural basis for functional divergence within the family.

Collectively, the three modules embody three functions, namely target recognition, signal integration, and transcriptional output. Their coordination allows BZR1/BES1 to convert diverse upstream signals into specific transcriptional programs. Crucially, this conversion is not automatic. It depends on the layered regulatory logic examined in the next section.

## 3. Multilayered Control of BZR1/BES1 Activity

### 3.1. The Canonical BIN2 Phosphorylation Switch and Its Counteracting Phosphatases

In the classical model, BIN2 is the central negative regulator of BZR1/BES1. When BR is scarce, active BIN2 phosphorylates multiple Ser/Thr residues in the central region of BZR1/BES1 [44]. At least twenty-five phosphosites have been mapped across the two proteins [15]. Phosphorylation promotes 14-3-3 binding, retains the factors in the cytoplasm, and ultimately targets them for degradation, thereby silencing BR-response genes [9,10]. When BR is abundant, the hormone is perceived by the extracellular LRR domain of BRI1 [45], driving BRI1–BAK1 heterodimerization and a downstream phosphorylation cascade [46] that sequentially activates BSK1 and CDG1 and, through them, the BSU1 family of phosphatases [47,48].

**Figure 2:**
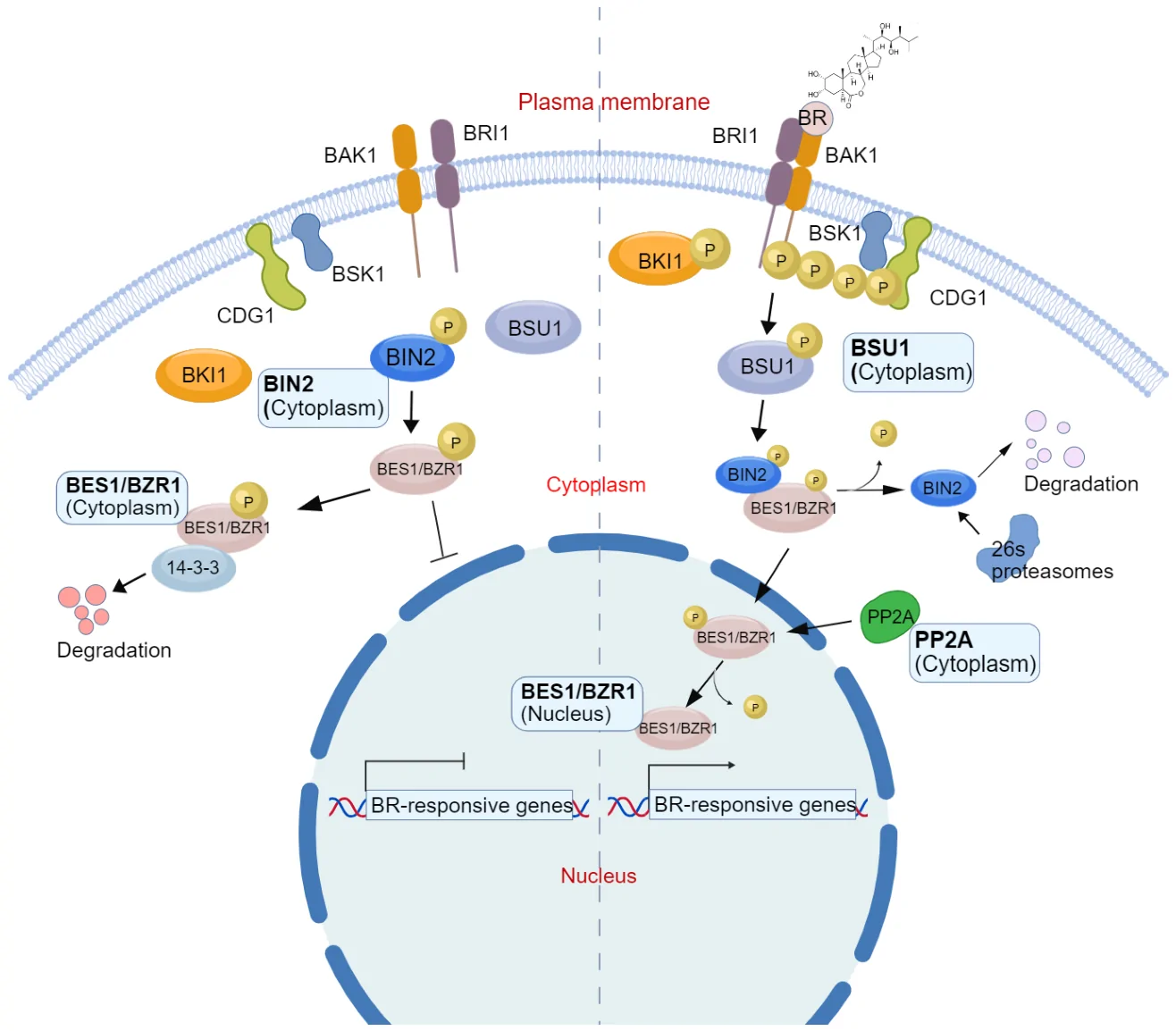
Overview of the brassinosteroid (BR) signaling pathway. In the absence of BR (**left**), the GSK3-like kinase BIN2 is active and phosphorylates BZR1/BES1, promoting their retention in the cytoplasm by 14-3-3 proteins and their degradation, so that BR-responsive genes remain off. In the presence of BR (**right**), the hormone is perceived by the BRI1–BAK1 receptor complex, activating a phosphorylation cascade (BSK1/CDG1) that switches on the BSU1 phosphatases. BSU1 inactivates BIN2, and PP2A then dephosphorylates BZR1/BES1, allowing them to accumulate in the nucleus and reprogram transcription. “P” denotes phosphorylation. Diagram templates were adapted from BioGDP (https://biogdp.com) [28].

Activated BSU1 dephosphorylates and inactivates BIN2 [8]. With BIN2 off, phosphatases such as PP2A dephosphorylate BZR1/BES1, releasing them from 14-3-3 proteins and allowing nuclear entry, where they activate or repress thousands of BR-response genes [7]. Phosphorylation of these factors is not the exclusive province of BIN2. MPK6 can directly phosphorylate BES1 to help balance growth and immunity [49], an early indication that BZR1/BES1 integrate inputs from kinases beyond the core pathway. The canonical relay is summarized in Figure 2.

It is worth noting that the BIN2–BZR1/BES1 switch does not operate in isolation, because BR-biosynthetic flux upstream and the miRNA-silencing machinery downstream both modulate its input and output. DET2 is the steroid 5α-reductase that catalyzes an early step of brassinolide biosynthesis, and by controlling BR supply it indirectly modulates the inhibitory phosphorylation of BZR1/BES1 by BIN2 [50]. ROT3 (CYP90C1) is the cytochrome P450 enzyme that catalyzes late-stage C-23 hydroxylation [51]. *ROT3* not only encodes a BR-biosynthetic enzyme but is also a direct transcriptional target of BZR1, through which it participates in the feedback regulation of BR homeostasis [37], and the ROT3 protein itself interacts directly with AGO1 at the endoplasmic reticulum [52]. AGO1 is the core effector of miRNA-mediated gene silencing, and its subcellular partitioning is negatively regulated by BR signaling. BR treatment reduces the endoplasmic-reticulum pool of AGO1, whereas the BR-deficient det2 mutant shows increased endoplasmic-reticulum localization of AGO1 [52]. BZR1 also directly represses the transcription of *AGO10*, the *AGO1* homolog that sequesters miR165/166 [53]. Thus, DET2, ROT3, and AGO1/AGO10 converge on the BZR1/BES1 network at the levels of BR synthesis, feedback regulation, and miRNA silencing, respectively, embedding the classical BIN2 phosphorylation switch within a broader “hormone synthesis–signal transduction–miRNA silencing” regulatory network.

### 3.2. Upstream Regulators of BIN2 and BZR1/BES1

The BIN2–BZR1/BES1 module forms the core switch, but a growing set of upstream regulators has transformed it from a single phosphorylation node into a multi-level control system. These regulators fall into two groups. The first acts on BIN2 and the second acts directly on BZR1/BES1 (summarized in Table 1).

Several regulators tune BIN2 itself. Scaffold proteins actively shape BR output by controlling BIN2 localization and substrate choice [55]. For example, in the stomatal lineage the scaffold POLAR and its homolog POLAR-LIKE1 (PL1) bind BIN2 and direct its activity toward stomatal-development substrates [54,55]. A microRNA module provides another layer. In rice, OsmiR159d does not target BZR1 directly but acts as a downstream response module that is itself suppressed by BR signaling, thereby linking BR and GA signaling through its target OsGAMYBL2 [70]. miR394 and its target LEAF CURLING RESPONSIVENESS (LCR) act upstream of BIN2 and BZR1/BES1. miR394 dampens BR output by promoting BIN2 accumulation and reducing BZR1/BES1 abundance, thereby limiting the expression of hypocotyl-elongation genes such as *PRE1*, *PRE5*, *PRE6*, and *EXP8* [56]. Cross-species studies reveal further diversification. In rice, the receptor-like cytoplasmic kinase OsSTRK1 modulates BR signaling by phosphorylating the BIN2 ortholog OsGSK2 (also known as OsSK22) [57], and in *Brassica napus* a BIN2-like 1 kinase interacts with HY5 and BZR1 to influence plant architecture and agronomic traits [58]. BIN2 function is thus broadly conserved but mechanistically diversified across species.

A second group of regulators acts directly on BZR1/BES1, delivering environmental, metabolic, and developmental information straight to the hub. BIL7 is a plasma-membrane- and nucleus-localized protein phosphorylated by BIN2. Once phosphorylated, BIL7 enters the nucleus, binds BZR1, and promotes its nuclear accumulation. This defines a membrane-to-nucleus route that couples membrane–protein dynamics directly to transcriptional control [12]. UPL3, a HECT-family E3 ubiquitin ligase, mediates BZR1 degradation under sugar-limiting conditions. When sugar is plentiful, the sugar–TOR pathway phosphorylates and inhibits UPL3 to stabilize BZR1, wiring carbon status into the BR network [13]. The molecular chaperone HSP90 physically associates with BZR1/BES1 to regulate their phosphorylation state and stability, a function required for accurate downstream gene expression [11]. HSP90 also links GA and BR signaling by interacting with both DELLA proteins and BZR1 to control DELLA abundance and BZR1-dependent transcription [59]. Finally, the nuclear-import factor IMB4 binds BZR1 and BES1, enhancing their nuclear accumulation and protecting them from 26S-proteasome-mediated degradation, thereby coupling nucleocytoplasmic transport to protein stability [14].

Taken together, from scaffold proteins and microRNAs to lineage-specific kinases, and from BIL7 and UPL3 to HSP90 and IMB4, these regulators build a multidimensional network that allows the core BIN2–BZR1/BES1 module to respond sensitively to developmental cues, nutritional status, and environmental stress, achieving precise control over growth and adaptation.

### 3.3. The Post-Translational Modification Network

Phosphorylation by BIN2 is the most classical direct PTM governing BZR1/BES1. BZR1/BES1 is also directly modified by ubiquitination, SUMOylation, and redox modification. In addition, acetylation-related regulation contributes to this broader regulatory network, but it acts on upstream or associated pathway components. These direct PTMs and indirect, pathway-level modifications do not act in isolation. Through synergy and antagonism, they form an interdependent network that jointly sets the subcellular localization, stability, and transcriptional activity of BZR1/BES1 [15]. We consider these direct and indirect modifications in turn and then the crosstalk logic that couples them.

#### 3.3.1. Ubiquitination Controls Protein Stability

Ubiquitination has emerged as a regulator of BR-signaling dynamics on a par with phosphorylation, controlling the abundance and activity of components at essentially every node of the pathway [71]. At the level of the core transcription factors, the protein abundance of BZR1/BES1 is dynamically regulated by ubiquitination and deubiquitination. PUB40 mediates 26S-proteasome-dependent degradation of phosphorylated BZR1 specifically in roots [60]. Under glucose starvation, UPL3 promotes autophagy-dependent degradation of BZR1, a process inhibited by glucose–TOR signaling [13]. In contrast, the deubiquitinating enzymes UBP12/UBP13 deubiquitinate and stabilize BES1, thereby promoting BR signaling [72]. UPL3 interacts with BZR1 in vitro and in vivo, and loss of UPL3 raises BZR1 abundance and accelerates warm-temperature hypocotyl elongation [73], while under sugar-replete conditions the sugar–TOR pathway represses UPL3 to stabilize BZR1 and optimize carbohydrate use [13]. The interplay of E3 ligases and deubiquitinases recurs at other nodes. The E3 ligase SGD1 ubiquitinates and stabilizes BRI1 to promote signaling [74], whereas UBP12/UBP13 protect BRI1 from vacuolar degradation [61].

Ubiquitination also mediates crosstalk between pathways. Under phosphate deficiency in rice, the E3 ligase OsPUB77 is induced and, by degrading the BR transcription factor OsBZR3, coordinates shoot architecture with nutrient-stress adaptation [63]. The U-box ligase OsPUB24 acts as a negative regulator of BR signaling by promoting 26S-proteasome-dependent degradation of OsBZR1. Because OsPUB24 stability is itself controlled by phosphorylation via the BIN2 ortholog OsGSK2, this arrangement establishes a feedback loop [62]. The degradation route can also switch with physiological state. Under normal conditions BZR1 is turned over by the 26S proteasome, whereas under starvation UPL3 directs it toward autophagy-dependent degradation to restrain growth [13,62]. A recently described tomato WD40-repeat protein, BIW (BZR1-interacting WD40-repeat protein), adds a further E3-associated node. BIW promotes BZR1 ubiquitination and degradation to dampen BR signaling, and BIN2-mediated phosphorylation of BIW at Ser144 is required for its stability and activity. This defines a “BIN2–BIW–BZR1” degradation route [64]. Together, these studies show that ubiquitination tunes the strength and integrative capacity of BR signaling in space and time through a diversified set of ligases and degradation pathways.

#### 3.3.2. SUMOylation Integrates Environmental Cues

SUMOylation is a key node at which BR signaling integrates environmental information, acting at multiple points in the network to balance growth against stress responses. At the transcription-factor level it regulates BZR1 and BES1 in opposite directions. BR treatment promotes BZR1 SUMOylation, which weakens the BZR1–BIN2 interaction, stabilizes nuclear BZR1, and promotes growth. Under salt stress the SUMO protease ULP1a deSUMOylates BZR1 in the cytoplasm, reducing its stability [66]. In contrast, SIZ1-mediated SUMOylation of BES1 at K302 destabilizes the protein and lowers its transcriptional activity, negatively regulating BR signaling [65]. This opposite-sign regulation of two closely related factors is a clear example of family-level functional divergence.

The balance between SUMOylation and deSUMOylation is itself under BR control. BR induces ULP1a degradation to allow SUMOylated BZR1 to accumulate, whereas the biosynthesis inhibitor brassinazole stabilizes ULP1a and drives BZR1 deSUMOylation [66]. SUMOylation also operates at the receptor level. BRI1 is SUMOylated in vivo. As temperature rises, the deSUMOylating isopeptidase Desi3a removes SUMO from BRI1, strengthening the BRI1–BIK1 interaction and promoting BRI1 endocytosis. This feeds temperature information into the pathway [75]. At the co-receptor level, SUMOylation of BAK1 at K367 precisely tunes its association with BRI1 without affecting its binding to PEPR1. Mutating this site strengthens BRI1 binding but leaves PEPR1 binding intact, identifying SUMOylation as a molecular switch that routes BAK1 between signaling pathways [76]. Beyond BR signaling, SIZ1 also functions in phosphate-starvation and drought responses, underscoring the role of SUMOylation as an integration node between environmental and hormonal signals [65,77,78]. Collectively, SUMOylation regulates nodes from the membrane receptor to nuclear transcription factors in a spatiotemporally specific manner, forming a molecular bridge between growth and stress adaptation.

#### 3.3.3. Redox Modification Provides a Rapid Stress Switch

Redox modification regulates BZR1 largely through reversible oxidation of cysteine residues and provides a fast route for stress signals to reach the transcription factor. The redox state of Cys63 is a key determinant of BZR1 activity. Under resting conditions, the thioredoxin TRXh5 keeps Cys63 reduced (C63-SH) and BZR1 activity low, but reactive oxygen species such as H_2_O_2_ oxidize Cys63 to sulfenic acid (C63-SOH). This oxidative modification enhances BZR1 transcriptional activity by strengthening its interaction with the partner transcription factors PIF4 and ARF6. The process is fully reversible, as TRXh5 can directly reduce oxidized BZR1 [67]. BZR1 thereby becomes a core node connecting H_2_O_2_ and thioredoxin-based redox signaling to the BR pathway, and redox modification becomes a molecular basis for exchanging information between BR and environmental signals.

Subsequent work broadened this picture. At the transcriptional level, full up-regulation of BZR1 target genes depends on environmental input, and H_2_O_2_-mediated oxidation enhances the BZR1–PIF4 interaction specifically, showing at the level of DNA binding and complex assembly how BZR1 transcriptional activation depends on the convergence of environmental and redox cues [68]. Redox control also reaches the upstream kinase. BIN2 is activated by oxidation of several cysteines (Cys59, Cys95, Cys99, and Cys162), and mutating these residues impairs normal BR output [79]. This demonstrates that redox signals act at multiple pathway nodes. The physiological relevance of redox regulation is evident in crops. Under low-nitrogen stress, ROS accumulation in wheat spikes disrupts redox homeostasis and accelerates floret degeneration, and exogenous epibrassinolide restores panicle energy metabolism and the ascorbate–glutathione cycle to scavenge ROS and alleviate degeneration [80]. Under copper toxicity in rice, BR signaling mitigates ROS accumulation and lignin deposition by integrating ROS homeostasis with transcriptional control of copper transporters [81]. In sum, a thiol-based switch centered on BZR1 Cys63 lets BR signaling sense and respond rapidly to redox fluctuations, coupling developmental signals to environmental stress and offering operable targets for improving stress resilience.

#### 3.3.4. Acetylation Links BR Output to Energy Status

Acetylation influences BR signaling indirectly rather than through direct modification of BZR1/BES1. The histone deacetylase HDA6 enhances BR output by deacetylating and inhibiting the upstream kinase BIN2, and this effect is significant only under sugar-limiting conditions, integrating acetylation-based regulation with energy signaling [69]. More broadly, chromatin-level acetylation dynamics are entwined with BZR1/BES1 function through their association with the TPL–HDA19 corepressor machinery, so that histone-acetylation state helps set the transcriptional output of the pathway [82]. Whether BZR1/BES1 themselves are directly acetylated or methylated, and with what consequence, remains to be established [15].

#### 3.3.5. Crosstalk and Integration of PTMs

The modifications above act combinatorically, through synergy, antagonism, and cascade logic, to determine BZR1/BES1 activity. Phosphorylation and ubiquitination form a classical degradation cascade centered on PUB40, a U-box E3 ligase and a principal executor of BZR1 turnover. When the receptor is inactive, BIN2 phosphorylates both BZR1 and PUB40, strengthening their interaction so that PUB40 preferentially recognizes phosphorylated BZR1, ubiquitinates it, and directs it to the 26S proteasome [60,83]. SUMOylation and ubiquitination, by contrast, act antagonistically and with member-specific outcomes. SUMOylation stabilizes BZR1 to promote growth but destabilizes BES1 and lowers its activity, again illustrating divergence between paralogues [65,66]. The dynamic balance between SUMOylation and ubiquitination sets the final abundance of BZR1, and this balance is timed by phosphorylation. BR-triggered inactivation of BIN2 lowers BZR1/BES1 phosphorylation, and the dephosphorylated, nuclear proteins are then stabilized jointly by SUMOylation and deubiquitination to drive downstream activation [84]. Layered onto these protein-level events, acetylation couples the network to energy status via HDA6–BIN2 [69]. The net effect of these synergistic, antagonistic, and cascading relationships is to make BZR1/BES1 a tunable integration point rather than a simple switch.

## 4. How Activated BZR1/BES1 Reprogram the Genome

Understanding how BZR1/BES1 are switched on is only half the story. Equally important is how the activated proteins reprogram the genome. Genome-wide mapping of BES1 targets defined a BR transcriptional network of more than a thousand genes and established BZR1/BES1 as master regulators that both activate and repress large gene sets [6]. Three features explain how a single factor achieves this dual, context-specific output. These are cis-element preference, recruitment of corepressor and chromatin-modifying machinery, and combinatorial partnerships with other transcription factors.

Cis-element preference underlies part of the activation-versus-repression decision. BZR1 binds the BRRE (CGTG(T/C)G) element and tends to repress its direct targets, including feedback repression of BR-biosynthesis genes. By contrast, BES1, generally as a heterodimer with BIM1, binds E-boxes (CANNTG) and tends to activate transcription [37,38]. Because the atypical bHLH domain reads DNA shape as well as sequence [39], promoter architecture and the local density and spacing of these elements bias whether a given gene is activated or repressed.

Corepressor and chromatin-modifying partners convert cis-element binding into a chromatin state. Through their C-termini, BZR1/BES1 recruit the corepressor TOPLESS (TPL) and the histone deacetylase HDA19 to repress target genes, coupling the transcription factors to histone-deacetylation-based silencing [41,42]. The BES1–TPL–HDA19 module shapes the BR transcriptional network and controls developmental transitions, including early seedling establishment [42,82]. Conversely, BZR1/BES1 promote gene activation by engaging histone-modifying enzymes that remove repressive marks. The Jumonji-domain H3K27 demethylases ELF6 and REF6 modulate BR-regulated gene expression as BES1 partners [85], and BR-activated BZR1 recruits ELF6 to the FLC locus to erase H3K27me3 [86]. Chromatin accessibility is a further control point. The BRAHMA-type SWI/SNF chromatin remodeler (BAS complex) is required for BR-induced transcriptional activation and growth, positioning nucleosome remodeling upstream of productive BZR1/BES1 output [87].

Combinatorial partnerships with other transcription factors provide the final layer of specificity, allowing BZR1/BES1 to fold environmental and hormonal information into their output. The best-characterized partner is PIF4. BZR1 and PIF4 interact physically and co-regulate roughly two thousand shared targets, and full elongation in response to BR, darkness, or warmth requires both factors [88]. This pair sits within a broader central circuit of interacting transcription factors (including ARF6, PIFs, and additional bHLH proteins) that integrates hormonal, light, and temperature cues to control hypocotyl cell elongation [89]. BR, GA, and phytochrome signals converge on this common transcription module, with DELLA proteins and PIFs acting alongside BZR1 to set growth [90]. Redox state gates these partnerships, as H_2_O_2_-dependent oxidation of BZR1 Cys63 strengthens its association with PIF4 and ARF6 [67,68]. Additional partners route BZR1/BES1 toward particular programs, such as BLH1 in thermomorphogenesis [91], STOP1 in phosphate-limited malate secretion [17], and NAC29/NAC31 in rice immunity and tillering [21]. In this view, the identity of the available partner, together with cis-element context and chromatin state, determines which subset of the BZR1/BES1 regulon is deployed in a given cell and condition.

## 5. Physiological Functions from Nutrient Sensing to Stress Adaptation

### 5.1. Nutrient Sensing and Metabolic Integration

Although first described as BR end-effectors, BZR1/BES1 are now known to integrate multiple nutrient signals, including carbon, nitrogen, phosphorus, sulfur, and boron, into growth and metabolic decisions.

Carbon and sugar status directly shape the balance between growth and stress adaptation. The HECT-type E3 ligase UPL3 mediates low-sugar-induced BZR1 degradation and is negatively controlled by the glucose–TOR pathway [13]. Sugar also promotes hypocotyl elongation in the dark through BR. Sugar treatment has little effect on BR biosynthesis but raises BZR1/BES1 transcript levels, maintains high BZR1 protein, and induces cell-elongation genes such as *IAA19*, *PRE1*, *PRE5*, *XTH18*, *EXPL2*, and *EXPA8* [92]. BR biosynthesis and signaling are thus required for sugar-promoted skotomorphogenic growth.

Nitrogen availability is integrated chiefly through BES1. Low nitrate up-regulates *BES1* transcription, especially in roots, and promotes accumulation of its dephosphorylated, active form. Activated BES1 directly induces nitrate-transporter genes *NRT2.1* and *NRT2.2* [16]. By antagonizing the repressor LBD37, it relieves inhibition of lateral-root growth [93]. In tomato, BR-activated BZR1 binds the promoters of the autophagy genes *ATG2* and *ATG6* to induce autophagy, reducing ubiquitinated-protein accumulation and alleviating nitrogen-starvation-induced chlorosis [94]. Over-expression of the BZR1 homolog *BEH4* improves nitrogen-use efficiency under low nitrogen by enhancing root growth and nitrogen-uptake gene expression [95]. In rice, BZR1 binds and activates the promoter of ammonium-transporter gene *AMT1;2* to promote ammonium uptake [96], while high ammonium enhances BR biosynthesis through a miR444–MADS–OsBRD1 cascade to restrain root elongation [97], revealing finely balanced growth control within the ammonium environment.

Phosphorus (Pi) integration links BR signaling to root nutrient foraging and iron homeostasis. Because soil Pi is often locked in iron- and aluminum-phosphate precipitates, plants secrete malate to mobilize it, and excess iron accumulates in Pi-starved roots. BZR1/BES1 restrain this low Pi-induced iron accumulation by directly repressing the ferroxidase gene *LPR1*, relieving inhibition of root elongation [98]. Pi deficiency also suppresses BR signaling and lowers *BZR1* expression. Conversely, exogenous BR or constitutively active bzr1-D reduces primary-root inhibition under Pi starvation by down-regulating *ALMT1* and malate secretion. This is because BZR1 competitively antagonizes the activation of *ALMT1* by STOP1. Therefore, the nuclear STOP1/BZR1 ratio sets *ALMT1* expression and malate efflux [17]. This logic is conserved. In rice, OsSPX1/2 interact with OsBZR1 under both Pi-sufficient and Pi-deficient conditions to repress BR-response genes and fine-tune the growth–immunity trade-off [27].

Sulfur is integrated through a BR–BZR1–SULTR module. Under sulfur deficiency, BR signaling is activated and BZR1 is up-regulated at both transcript and protein levels and accumulates in the nucleus. BZR1 then directly targets the high-affinity sulfate transporter *SULTR1;2* to promote sulfate uptake in a BR-dependent manner [18].

Boron is subject to bidirectional regulation by the family. Boron deficiency represses the BR-biosynthesis genes *BR6ox1*/*BR6ox2*, weakening BR signaling and BR-dependent root growth [99]. By contrast, boron toxicity up-regulates *DWF4* and *BR6ox1*/*2* and represses the inactivating gene *BAS1*, sustaining BR signaling. Activated BZR1/BES1 then cooperate with PIF4 to drive hypocotyl elongation [19].

These examples show that BZR1/BES1 coordinate metabolic adaptation and growth across contrasting nutrient regimes. A key open problem is that most studies address one nutrient at a time, whereas plants face simultaneous, fluctuating changes in many nutrients. How different nutrient signals are discriminated and weighted on the same BZR1/BES1 molecule within one cell, and whether they act synergistically or antagonistically, remains unclear. Multi-omics and systems approaches that resolve BZR1/BES1 target networks under combined nutrient conditions will be essential.

### 5.2. Yield-Related Developmental Traits

**Figure 3:**
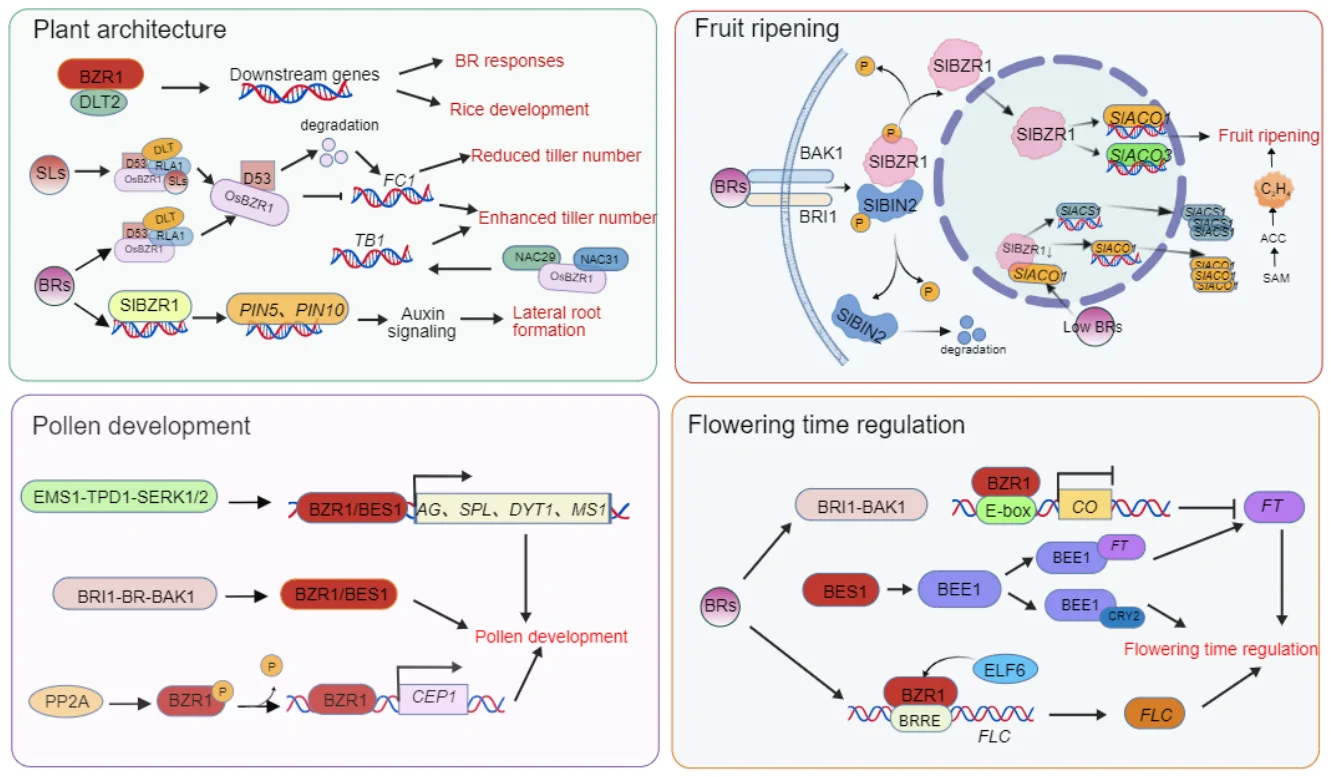
Roles of BZR1/BES1 in yield-related developmental traits. BZR1/BES1 and their crop orthologs regulate plant architecture (tillering and branching, root growth), fruit ripening, pollen development, and flowering time, integrating BR signals with developmental and environmental cues to shape agronomically important traits. Diagram templates were adapted from BioGDP (https://biogdp.com) [28].

As the core BR transcription factors, BZR1/BES1 extend well beyond cell elongation and photomorphogenesis to control agronomically important traits, including plant architecture, fruit ripening, pollen development, and flowering time (Figure 3).

#### 5.2.1. Plant Architecture

BZR1/BES1 play an important role in shaping shoot and root architecture. In rice, BR signaling promotes tiller outgrowth and acts together with strigolactones through the D53–OsBZR1–RLA1–DLT module, with FINE CULM 1 (FC1/OsTB1) acting downstream to suppress tillering [100,101]. The GRAS-family factor DLT2 (DWARF AND LOW-TILLERING 2) interacts with OsBZR1 and DLT to assemble BR-signaling transcription complexes that shape rice development [102]. More recently, a BZR1–NAC29–NAC31 module was shown to activate the defense gene *CAD8B* while repressing the tillering suppressor *TB1*, thereby coupling enhanced disease resistance to increased tiller number [21]. In the dicot *Medicago truncatula*, the *mtbzr1* mutant shows markedly reduced branching and biomass. MtBZR1 promotes axillary-bud outgrowth both by enhancing photosynthetic capacity and by cooperating with hormone signaling to increase auxin export and to repress *MtBRC1*. Its over-expression in alfalfa raises branch number and forage yield [103]. BZR1 also controls root architecture. In tomato, lateral-root formation is impaired in BR-biosynthesis (*dwf*) and signaling (*bzr1*) mutants, whereas exogenous BR or *BZR1* over-expression increases lateral-root density by up-regulating the auxin-transporter genes *PIN5* and *PIN10* [104]. Thus, BZR1-dependent repression of branch-inhibitory genes such as *FC1*/*OsTB1* and *MtBRC1* is conserved for shoot branching in monocots and dicots, whereas BZR1-regulated root development relies on distinct downstream mechanisms, for example auxin-transporter-mediated pathways. Notably, the BZR1–NAC29–NAC31 module suggests that editing specific BZR1 partners, rather than BZR1 itself, could enhance tillering while preserving defense, which is an attractive breeding strategy.

#### 5.2.2. Fruit Ripening

BZR1/BES1 regulate fruit ripening in a species-specific manner. In tomato, SlBZR1 is a principal positive regulator that promotes ripening and carotenoid accumulation by directly activating the ethylene-biosynthesis genes *SlACO1* and *SlACO3* and the carotenoid gene *SlPSY1* [105]. Consistent with this, over-expression of *AtBES1-D* in tomato enhances BR response and accelerates ripening while improving fruit quality through coordinated effects on BR, ethylene, transcription-factor, and metabolic networks [22]. Other family members act in related processes. SlBES2 contributes to photosynthetic-apparatus development in tomato fruit [106], and BZR1.3 controls fruit size by recruiting the H3R2me2a demethylase JMJ10 [107]. In climacteric tree fruits such as apple and pear, by contrast, BR-activated BZR1 represses ripening by inhibiting the ethylene-biosynthesis genes *ACO1* and *ACS1*, lowering ethylene production and improving storability [108]. The opposite effects of orthologous factors in different species, likely reflecting differences in promoter cis-element context and interacting partners, offer contrasting breeding levers. One can enhance BZR1 activity to accelerate ripening and quality in tomato, or maintain BZR1-mediated repression to extend shelf life in pome fruits.

#### 5.2.3. Pollen Development

BZR1/BES1 are indispensable for male reproductive development, with strong functional redundancy among the six family members [31]. Single *bes1* or *bzr1* mutants show no obvious pollen-wall defects, but *bes1 bzr1* double and higher-order mutants display pollen-wall abnormalities [31,109]. A PP2A–BZR1–CEP1 module underlies part of this control. The tapetum-expressed PP2A B′α and B′β subunits dephosphorylate and activate BZR1, which then directly binds the promoter of the cysteine-protease gene *CEP1* to activate its transcription. The gain-of-function bzr1-1D allele restores fertile pollen in a B′α/B′β-dependent manner [109]. BZR1/BES1 also activate anther-development genes such as *AG*, *SPL*, *DYT1*, and *MS1* [20], and gain-of-function bes1-D and bzr1-1D partially rescue the tapetal defects and male sterility of the *tpd1* mutant [31]. Furthermore, the receptor-like kinases EMS1 and BRI1 jointly regulate filament elongation through BES1/BZR1 [110]. Two themes emerge. First, family redundancy means only higher-order mutants reveal the essential requirement. Second, anther BZR1 activation is driven largely by the EMS1–TPD1–SERK1/2 pathway rather than solely by BRI1 [31]. This is a BR-receptor-independent input consistent with the hub role of BZR1/BES1.

#### 5.2.4. Flowering Time

BZR1/BES1 exert dual, context-dependent control over flowering. On the one hand, BES1 promotes photoperiodic flowering indirectly. Unable to bind the *FT* promoter directly, BES1 acts through its direct target *BEE1*, which binds *FT* chromatin to activate transcription and also interacts with the blue-light receptor CRY2 to integrate light signals [111]. On the other hand, BR signaling can delay flowering. Under long days, BZR1 binds the *CONSTANS* (*CO*) promoter and represses its transcription, acting upstream of CO and FT to postpone flowering according to day length [23]. In the presence of BR, BZR1 also binds the BRRE element in the *FLC* intron and recruits the H3K27 demethylase ELF6 to remove repressive H3K27me3, up-regulating the floral repressor FLC and thereby inhibiting the floral transition [86]. By selectively targeting *FT*, *CO*, and *FLC*, BZR1/BES1 act as an integration point between endogenous BR signals and photoperiodic cues, providing molecular levers for tuning flowering time in crops.

### 5.3. Abiotic-Stress Tolerance and Osmotic/Redox Homeostasis

**Figure 4:**
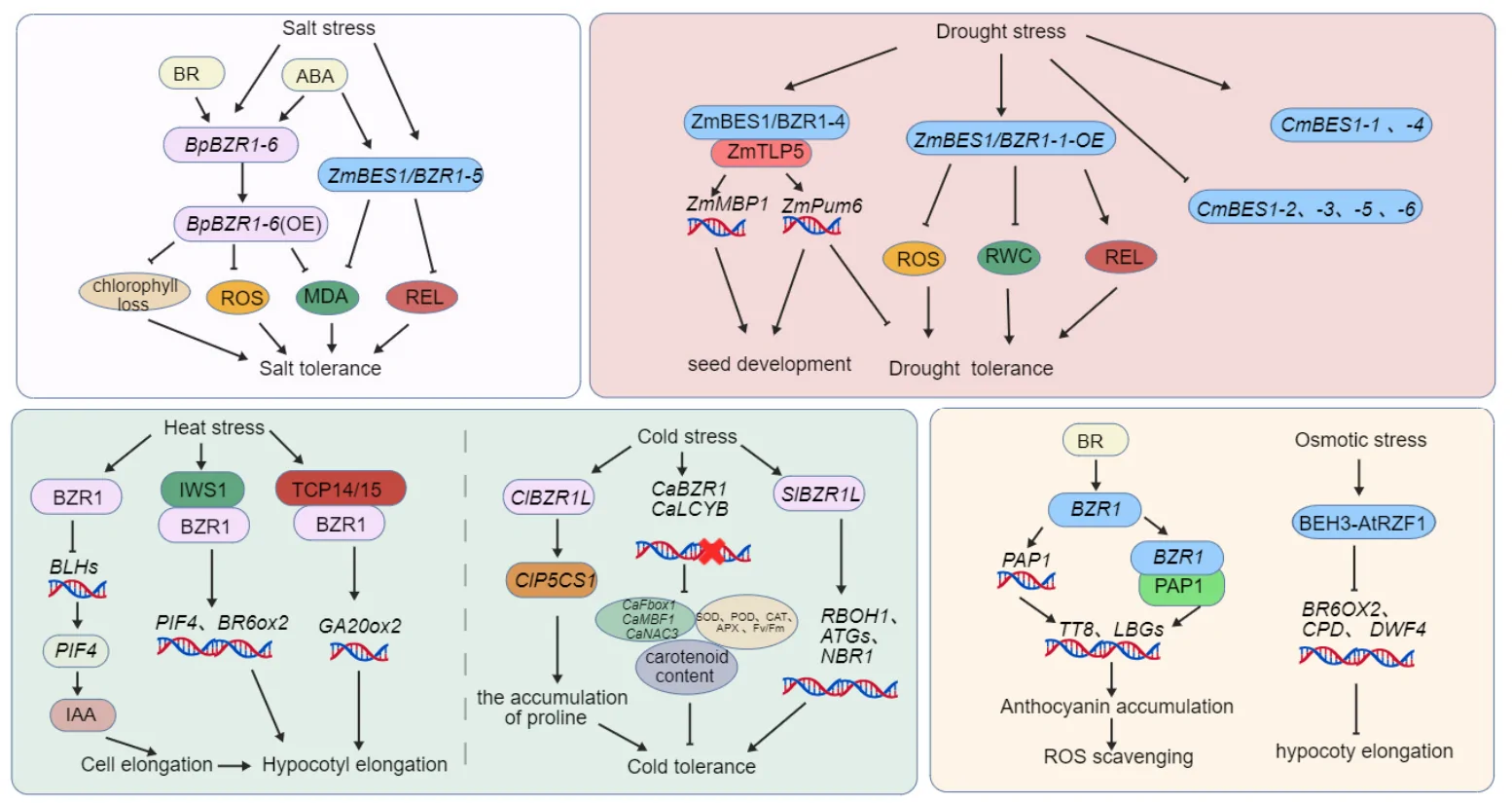
Roles of BZR1/BES1 in abiotic-stress tolerance and cellular homeostasis. Across species, BZR1/BES1 modulate responses to salinity, drought, and temperature extremes, and contribute to osmotic adjustment and reactive-oxygen-species (ROS) scavenging, largely by regulating antioxidant and stress-responsive genes. Arrows indicate the direction of regulatory effects between components and the red X superimposed on the DNA symbol indicates inhibition of transcription. Diagram templates were adapted from BioGDP (https://biogdp.com) [28].

BZR1/BES1 have well-documented roles in abiotic-stress tolerance and cellular homeostasis, extending from Arabidopsis to diverse crops and converging on ROS scavenging and stress-gene regulation (Figure 4).

#### 5.3.1. Salt Tolerance

A positive role for BZR1/BES1 in salt tolerance has been validated in cross-species heterologous assays and typically involves ROS detoxification and downstream target regulation. In birch (*Betula platyphylla*), *BpBZR1-6* is induced by salt, ABA, and BR in the native species. When heterologously expressed in Arabidopsis, it enhances salt tolerance by boosting antioxidant-enzyme gene expression and ROS-scavenging capacity [24]. Maize *ZmBES1/BZR1-5* is likewise induced by ABA, salt, and drought in maize. Its heterologous expression in Arabidopsis reduces ABA sensitivity, promotes shoot and root growth, and lowers malondialdehyde content and relative electrolyte leakage under osmotic stress, enhancing both salt and drought tolerance in the heterologous system [112].

#### 5.3.2. Drought Tolerance

Drought responses reveal clear functional differentiation among family members. In maize, *ZmBES1*/*BZR1-5* is induced by drought, and its heterologous expression in Arabidopsis enhances drought tolerance [112]. Maize ZmBES1/BZR1-4 directly binds to E-box elements in the *ZmPum6* and *ZmMBP1* promoters to activate their transcription, and its interaction with ZmTLP5 enhances this transcriptional activation. Overexpression of *ZmBES1*/*BZR1-4* in transgenic Arabidopsis, rice, and maize reduced drought tolerance but increased seed size and weight [25], whereas ZmBES1/BZR1-1 acts as a negative regulator of drought tolerance in maize [113]. Genome-wide transcriptional analysis in melon similarly shows differentiated transcriptional responses of CmBES1 members to drought and cold [114]. This division of labor indicates that the net effect of the family on drought depends on the specific member, tissue, and stress intensity.

#### 5.3.3. Temperature Stress

BZR1/BES1 act bidirectionally in temperature adaptation. Under high temperature, studies in Arabidopsis show that BZR1 represses the negative regulator *BLH1* (*BEL1-like homeodomain 1*), relieving BLH1-mediated repression of the growth-promoting factor PIF4. This BZR1–BLH1–PIF4 pathway feeds a BZR1–PIF4–auxin–BR positive-feedback loop to ensure a balanced thermomorphogenic response [91]. In parallel, the transcription-elongation factor IWS1 (Interact-With-SPT6) promotes thermomorphogenesis through two modules. Warm temperatures enhance IWS1 chromatin association. The IWS1–BZR1 complex then binds the *PIF4* promoter in a temperature-dependent manner to enhance its transcription and up-regulates the BR-biosynthesis gene *BR6ox2*, while IWS1 also engages the GA-related factors TCP14/15 to activate *GA20ox2* and promote DELLA degradation, relieving GA-mediated growth restraint [115]. Under low temperature, BZR1/BES1 are core regulators of cold tolerance but act through species-specific downstream effectors. In watermelon, ClBZR1L binds and activates the proline-biosynthesis gene *ClP5CS1* to accumulate proline and maintain osmotic balance [26]. In pepper, CaBZR1 enhances cold tolerance via the carotenoid gene *CaLCYB*, raising antioxidant capacity and ROS scavenging [116]. In tomato, SlBZR1 confers cold tolerance by targeting *RBOH1* [117] and the autophagy machinery, namely ATGs and NBR1 [118]. BZR1/BES1 thus mediate responses to both temperature extremes through multiple, partly species-specific mechanisms.

#### 5.3.4. Osmotic Homeostasis and ROS Scavenging

Maintaining osmotic balance and limiting ROS accumulation are central to abiotic-stress resistance, and BZR1/BES1 contribute on both fronts. When heterologously expressed in Arabidopsis, BpBZR1-6 enhances ROS-scavenging capacity by boosting antioxidant-enzyme activity [24]. In Arabidopsis, BZR1 also promotes anthocyanin biosynthesis by binding the *PAP1* promoter and interacting with the PAP1 protein [119]. As antioxidants, anthocyanins support ROS scavenging and photoprotection under stress [120]. Family members can also act negatively. The Arabidopsis homolog BEH3 cooperates with the E3 ligase AtRZF1 in an AtRZF1–BEH3 pathway as a negative regulator of the osmotic stress responses, and BEH3 additionally suppresses dark hypocotyl elongation by repressing the BR-biosynthesis genes *BR6ox2*, *CPD*, and *DWF4* [121]. Overall, BZR1/BES1 provide candidate molecular targets for improving stress resilience through gene editing and molecular breeding. However, because many crop BZR1/BES1 functions are currently inferred from heterologous expression in Arabidopsis, these findings establish functional potential and candidate targets rather than native crop functions, which will require species-specific validation.

### 5.4. Dedifferentiation, Pluripotency, and Tissue Culture

Beyond their roles in intact plants, BZR1/BES1 also help govern the balance between pluripotency and differentiation, a dimension directly relevant to plant tissue culture in which brassinosteroids are widely used. In Arabidopsis, BZR1 promotes pluripotency acquisition and callus development by directly activating the auxin-response factors ARF7 and ARF19, two master regulators of pluripotency [122]. BES1 can instead restrain callus formation. The BES1–KCS1 module up-regulates very-long-chain fatty acid (VLCFA) biosynthesis, and loss of BES1 or KCS1 increases callus formation, indicating that BES1 quantitatively suppresses pluripotent callus development through KCS1 [123]. In the root stem-cell niche, paracrine BR signaling through BES1 promotes quiescent-center divisions to replenish stem cells after DNA damage [124], and BZR1-dependent signaling stimulates ectopic quiescent-cell division while suppressing columella stem-cell differentiation [125]. Together, these findings position BZR1/BES1 as regulators of pluripotency and dedifferentiation, with practical implications for optimizing regeneration and transformation protocols in tissue-culture systems.

### 5.5. Cross-Species Functional Divergence

A recurring theme across the preceding sections is that BZR1/BES1 orthologs, though built on a conserved core, have been extensively rewired during evolution, and that much of our mechanistic knowledge still derives from Arabidopsis (Table 2). Several patterns stand out. First, the core architecture and the BIN2-mediated switch are conserved, yet lineage-specific regulators have been recruited to reshape the module in each species. These include kinases such as rice OsSTRK1, which phosphorylates OsGSK2 [57], E3 ligases such as rice OsPUB24 and OsPUB77 [62,63], and the tomato WD-repeat protein BIW [64]. Second, the same trait can be regulated in opposite directions in different species. BZR1 promotes fruit ripening in tomato but represses it in apple and pear [105,108]. Third, within a single crop the family has diversified into positive and negative regulators of the same stress, as seen for maize ZmBES1/BZR1 members in drought [25,112,113]. Fourth, crop orthologs have acquired distinct nutrient- and immunity-integration roles. For example, rice OsBZR1 couples BR signaling to phosphate status and pathogen defense through OsSPX1/2 and NAC29/NAC31 [21,27]. This divergence means that findings from Arabidopsis cannot be assumed to transfer directly to crops, and it argues for systematic, species-resolved functional dissection using genome editing and ortholog-specific study.

## 6. Conclusions and Future Perspectives

The reconceptualization of BZR1/BES1 from terminal effectors of the BR pathway into multi-signal integration hubs for growth and stress adaptation is among the more consequential shifts in plant hormone signaling in recent years. Structurally, the modular architecture of these proteins enables simultaneous target recognition, signal input, and transcriptional output. Mechanistically, the canonical BIN2 phosphorylation switch is now embedded in a much larger network. Direct regulators such as BIL7, UPL3, HSP90, and IMB4 couple membrane dynamics, carbon status, protein homeostasis, and nuclear import to BZR1/BES1, while ubiquitination, SUMOylation, redox modification, and acetylation form an interdependent post-translational code that raises regulation from a linear relay to a tunable, multidimensional system. At the point of transcriptional execution, cis-element preference, corepressor and chromatin-modifying partners, and combinatorial associations with factors such as PIF4 and ARF6 allow the same proteins to deploy distinct sub-programs in different contexts. Physiologically, this integrative capacity manifests as coordinated control of nutrient-responsive metabolism, yield-related development, and abiotic-stress tolerance, and as a central role in arbitrating growth–defense trade-offs.

Several challenges remain. First, most studies still examine a single nutrient or a single stress, whereas plants integrate many simultaneous inputs. We lack a systematic account of how distinct signals converge on, and are discriminated by, the same BZR1/BES1 molecule, and of how particular PTM combinations produce specific outputs. This post-translational code is the temporal and spatial logic by which phosphorylation, ubiquitination, SUMOylation, redox modification, and acetylation jointly set the stability, localization, and activity of BZR1/BES1, and decoding it is a priority. Second, translating mechanism into crop improvement is complicated by the pleiotropy that accompanies constitutive over-expression or knockout of these hubs. Two strategies are promising. The first is to identify condition-, tissue-, and stage-specific BZR1/BES1 partners and target genes and to edit those downstream components rather than BZR1/BES1 themselves, as suggested by the BZR1–NAC29–NAC31 module. The second is to use CRISPR–Cas9 cis-regulatory editing to fine-tune *BZR1*/*BES1* expression under defined conditions, preserving core growth functions while enhancing resilience. Third, because crop orthologs have diverged substantially from Arabidopsis, systematic functional work in major crops, combining CRISPR-based allelic series, GWAS, and tissue-specific or chemically inducible expression systems, is needed to convert model-plant knowledge into breeding gains.

Looking ahead, integrating AI-assisted structure prediction, multi-omics, in vivo imaging, and synthetic-biology reconstruction should enable a predictive understanding that links molecular events to physiological outputs. A particularly promising frontier is transcriptional condensation. BZR1/BES1 family members contain predicted intrinsically disordered regions, and liquid–liquid phase separation is increasingly implicated in plant signal integration. This raises the possibility that condensate formation contributes to signal-specific output, although no direct experimental evidence for BZR1/BES1 condensates exists yet, making this a speculative but testable frontier. We therefore propose this as a hypothesis and an important direction for future work. If experimentally validated, condensate formation could represent a potential, not yet proven, target for tuning architecture, yield, and stress resistance.

## Figures and Tables

**Table 1:** Upstream and post-translational regulators of BZR1/BES1.

Regulator *	Type	Modification/Action	Effect on BZR1/BES1	Species	Reference
BIN2 (GSK3-like)	Kinase	Phosphorylates Ser/Thr sites in central region	Cytoplasmic retention, degradation, reduced DNA binding	Arabidopsis	[15,44]
BSU1 family	Phosphatase	Dephosphorylates and inactivates BIN2	Relieves inhibition	Arabidopsis	[8]
PP2A	Phosphatase	Dephosphorylates BZR1/BES1	Releases from 14-3-3, promotes nuclear entry	Arabidopsis	[35]
POLAR/PL1	Scaffold	Bind BIN2, direct its activity to stomatal substrates	Spatially targets BIN2 activity	Arabidopsis	[54,55]
miR394/LCR	miRNA module	Acts upstream of BIN2, promotes BIN2 accumulation	Reduces BZR1/BES1 abundance	Arabidopsis	[56]
OsSTRK1	Kinase (RLCK)	Phosphorylates BIN2 ortholog OsGSK2	Modulates BR signaling	Rice	[57]
BnBIL1	Kinase	Interacts with HY5 and BZR1	Influences architecture/agronomic traits	Brassica napus	[58]
BIL7	Membrane/nuclear protein	BIN2-phosphorylated, binds BZR1 in nucleus	Promotes nuclear accumulation	Arabidopsis	[12]
UPL3	E3 ligase (HECT)	Ubiquitinates BZR1; repressed by sugar–TOR	Degradation, couples carbon status	Arabidopsis	[13]
HSP90	Chaperone	Associates with BZR1/BES1, binds DELLA	Regulates phosphorylation state and stability; links GA–BR	Arabidopsis	[11,59]
IMB4	Nuclear-import factor	Binds BZR1/BES1	Enhances nuclear accumulation, protects from 26S degradation	Arabidopsis	[14]
PUB40	E3 ligase (U-box)	Recognizes BIN2-phosphorylated BZR1, ubiquitinates it	26S-proteasomal degradation	Arabidopsis	[60]
UBP12/UBP13	Deubiquitinase	Remove ubiquitin from BZR1/BRI1	Counter-stabilize	Arabidopsis	[61]
OsPUB24	E3 ligase (U-box)	Promotes OsBZR1 degradation	Negative regulator	Rice	[62]
OsPUB77	E3 ligase	Induced by Pi deficiency; degrades OsBZR3	Couples shoot architecture to nutrient stress	Rice	[63]
BIW	WD40 protein (E3-associated)	BIN2-phosphorylated at Ser144; promotes BZR1 ubiquitination	Degradation (“BIN2–BIW–BZR1” route)	Tomato	[64]
SIZ1	SUMO E3 ligase	SUMOylates BES1	Destabilizes BES1; lowers activity	Arabidopsis	[65]
ULP1a	SUMO protease	DeSUMOylates BZR1 in cytoplasm	Reduces BZR1 stability	Arabidopsis	[66]
TRXh5	Thioredoxin	Reduces oxidized BZR1 Cys63	Keeps BZR1 activity low	Arabidopsis	[67]
H_2_O_2_/ROS	Redox signal	Oxidize BZR1 Cys63 to sulfenic acid	Strengthens PIF4/ARF6 binding	Arabidopsis	[67,68]
HDA6	Histone deacetylase	Deacetylates and inhibits BIN2	Enhances BR output	Arabidopsis	[69]

* Regulators are grouped by the layer at which they act (core phosphorylation switch, regulators of BIN2, direct regulators of BZR1/BES1, and the ubiquitination, SUMOylation, redox, and acetylation nodes of the post-translational network).

**Table 2:** Cross-species BZR1/BES1 family members and their documented functions.

Species	Gene(s)	Primary Documented Function(s)	Direction of Effect *	Reference
Arabidopsis	BZR1	Cell elongation; BR-gene repression via BRRE; flowering (CO, FLC); thermomorphogenesis	Context-dependent (mainly positive for growth)	[23,37,91]
Arabidopsis	BES1	BR-gene activation via E-box (with BIM1); pollen; growth–defense balance	Positive (growth)	[6,38]
Arabidopsis	BEH3	Osmotic-stress response (with AtRZF1); represses BR biosynthesis	Negative (osmotic stress)	[121]
Rice	OsBZR1	Tillering; couples BR to phosphate status and immunity (OsSPX1/2, NAC29/31)	Positive (context-dependent)	[21,27]
Rice	OsBZR3	Shoot architecture under phosphate deficiency (OsPUB77 target)	Regulated by nutrient stress	[63]
Maize	ZmBES1/BZR1-5	Salt and drought tolerance; ABA response (BAM-domain homodimer)	Positive (stress tolerance)	[36,112]
Maize	ZmBES1/BZR1-4	Drought tolerance and seed development (recruits ZmTLP5)	Positive (drought)	[25]
Maize	ZmBES1/BZR1-1	Drought response	Negative (drought)	[113]
Tomato	SlBZR1	Fruit ripening; cold tolerance (RBOH1, ATGs, NBR1)	Positive (ripening, cold)	[105,117,118]
Apple/Pear	MdBZR1/PbBZR1	Fruit ripening	Negative (represses ripening)	[108]
Watermelon	ClBZR1L	Cold tolerance via proline biosynthesis (ClP5CS1)	Positive (cold)	[26]
Pepper	CaBZR1	Cold tolerance via carotenoid gene CaLCYB (antioxidant/ROS)	Positive (cold)	[116]
Birch	BpBZR1-6	Salt/ABA/BR-induced; antioxidant enzymes, ROS scavenging	Positive (salt)	[24]
Melon	CmBES1 (family)	Differentiated transcriptional responses to drought and cold	Member-specific	[114]

* Orthologs share a conserved core but have been extensively rewired, so that the same trait can be regulated in opposite directions in different species and, within one crop, distinct family members can act as positive or negative regulators of the same process.

## Data Availability

No new data were created or analyzed in this study. Data sharing is not applicable to this article.

## Abbreviations

The following abbreviations are used in this manuscript:

ABA	abscisic acid
ACC	1-aminocyclopropane-1-carboxylic acid
ACO	ACC oxidase
ACS	ACC synthase
AG	AGAMOUS
AGO1	ARGONAUTE1
AGO10	ARGONAUTE10
AI	artificial intelligence
ALMT1	ALUMINUM-ACTIVATED MALATE TRANSPORTER 1
AMT1;2	AMMONIUM TRANSPORTER 1;2
ARF6	AUXIN RESPONSE FACTOR 6
ARF7/19	AUXIN RESPONSE FACTOR 7/19
ATG	AUTOPHAGY-RELATED
AtRZF1	ARABIDOPSIS THALIANA RING ZINC FINGER 1
BAK1	BRI1-ASSOCIATED RECEPTOR KINASE 1
BAS	BRAHMA-type SWI/SNF chromatin-remodeling complex
BAS1	PHYB ACTIVATION-TAGGED SUPPRESSOR 1
BEE1	BR-ENHANCED EXPRESSION 1
BEH	BES1/BZR1 HOMOLOGUE
BES1	BRI1-EMS-SUPPRESSOR 1
bHLH	basic helix–loop–helix
BIK1	BOTRYTIS-INDUCED KINASE 1
BIM1	BES1-INTERACTING MYC-LIKE 1
BIN2	BR-INSENSITIVE 2
BIW	BZR1-INTERACTING WD40-REPEAT PROTEIN
BLH1	BEL1-LIKE HOMEODOMAIN 1
BR	brassinosteroid
BR6ox	BRASSINOSTEROID-6-OXIDASE
BRRE	brassinosteroid-response element
BSK1	BR SIGNALING KINASE 1
BSU1	BRI1 SUPPRESSOR 1
BZR1	BRASSINAZOLE-RESISTANT 1
CAD8B	CINNAMYL ALCOHOL DEHYDROGENASE 8B
Cas9	CRISPR-associated protein 9
CBF	C-REPEAT BINDING FACTOR
CDG1	CONSTITUTIVE DIFFERENTIAL GROWTH 1
CEP1	CYSTEINE PROTEASE 1
CO	CONSTANS
CPD	CONSTITUTIVE PHOTOMORPHOGENESIS AND DWARFISM
CRISPR	clustered regularly interspaced short palindromic repeats
CRY2	CRYPTOCHROME 2
CYP90C1	cytochrome P450 90C1
D53	DWARF 53
Desi3a	deSUMOylating isopeptidase 3a
DET2	DE-ETIOLATED 2
DLT	DWARF AND LOW-TILLERING
DWF4	DWARF 4
DYT1	DYSFUNCTIONAL TAPETUM 1
E-box	enhancer box
ELF6	EARLY FLOWERING 6
EMS1	EXCESS MICROSPOROCYTES 1
EXP	expansin
FC1	FINE CULM 1
FLC	FLOWERING LOCUS C
FT	FLOWERING LOCUS T
GA	gibberellin
GRAS	GIBBERELLIC ACID INSENSITIVE/REPRESSOR OF GAI/SCARECROW
GSK3	GLYCOGEN SYNTHASE KINASE 3
GWAS	genome-wide association study
H3K27me3	histone H3 lysine 27 trimethylation
HDA	histone deacetylase
HECT	homologous to E6-AP C-terminus
HSP90	heat shock protein 90
HY5	ELONGATED HYPOCOTYL 5
IAA19	INDOLE-3-ACETIC ACID 19
IMB4	IMPORTIN BETA 4
IWS1	INTERACT WITH SPT6
JMJ10	JUMONJI 10
KCS1	3-KETOACYL-COA SYNTHASE 1
LBD37	LATERAL ORGAN BOUNDARIES DOMAIN 37
LCR	LEAF CURLING RESPONSIVENESS
LCYB	LYCOPENE BETA-CYCLASE
LPR1	LOW PHOSPHATE ROOT 1
LRR	leucine-rich repeat
MADS	MCM1/AGAMOUS/DEFICIENS/SRF
miR	microRNA
MPK6	MITOGEN-ACTIVATED PROTEIN KINASE 6
MS1	MALE STERILITY 1
NAC	NAM/ATAF/CUC transcription factor
NBR1	NEIGHBOR OF BRCA1 1
NRT2	NITRATE TRANSPORTER 2
P5CS1	Δ1-PYRROLINE-5-CARBOXYLATE SYNTHASE 1
PAP1	PRODUCTION OF ANTHOCYANIN PIGMENT 1
PEPR1	PEP RECEPTOR 1
PEST	proline-, glutamate-, serine-, and threonine-rich
Pi	inorganic phosphate
PIF4	PHYTOCHROME-INTERACTING FACTOR 4
PIN	PIN-FORMED
PL1	POLAR-LIKE1
PP2A	protein phosphatase 2A
PRE	PACLOBUTRAZOL RESISTANCE
PSY1	PHYTOENE SYNTHASE 1
PTM	post-translational modification
PUB	PLANT U-BOX
RBOH1	RESPIRATORY BURST OXIDASE HOMOLOG 1
REF6	RELATIVE OF EARLY FLOWERING 6
RLCK	receptor-like cytoplasmic kinase
RNA-seq	RNA sequencing
ROS	reactive oxygen species
ROT3	ROTUNDIFOLIA3
SERK	SOMATIC EMBRYOGENESIS RECEPTOR KINASE
SIZ1	SAP AND MIZ1 DOMAIN-CONTAINING LIGASE 1
SPL	SPOROCYTELESS
STOP1	SENSITIVE TO PROTON RHIZOTOXICITY 1
SULTR	sulfate transporter
SUMO	small ubiquitin-like modifier
SWI/SNF	switch/sucrose non-fermentable
TB1	TEOSINTE BRANCHED 1
TCP	TEOSINTE BRANCHED1/CYCLOIDEA/PCF
TOR	target of rapamycin
TPD1	TAPETUM DETERMINANT 1
TPL	TOPLESS
TRXh5	thioredoxin h5
UBP	ubiquitin-specific protease
ULP1a	UBL-specific protease 1a
UPL3	UBIQUITIN-PROTEIN LIGASE 3
VLCFA	very-long-chain fatty acid
WD40	tryptophan–aspartate 40 repeat
XTH	xyloglucan endotransglucosylase/hydrolase
